# Tourette syndrome research highlights 2015

**DOI:** 10.12688/f1000research.8769.1

**Published:** 2016-06-24

**Authors:** Cheryl A. Richards, Kevin J. Black

**Affiliations:** 1 Department of Psychiatry, Washington University School of Medicine, St. Louis, Missouri, USA; 2 Department of Medicine, Washington University School of Medicine, St. Louis, Missouri, USA; 3 Department of Neurology, Washington University School of Medicine, St. Louis, Missouri, USA; 4 Department of Radiology, Washington University School of Medicine, St. Louis, Missouri, USA; 5 Department of Neuroscience, Washington University School of Medicine, St. Louis, Missouri, USA

**Keywords:** Tourette syndrome, tic disorders, genetics, animal models, neuroimaging, premonitory, therapy, review

## Abstract

We present selected highlights from research that appeared during 2015 on Tourette syndrome and other tic disorders. Topics include phenomenology, comorbidities, developmental course, genetics, animal models, neuroimaging, electrophysiology, pharmacology, and treatment. We briefly summarize articles whose results we believe may lead to new treatments, additional research or modifications in current models of TS.

## Introduction

This article is the second in the TS Research Highlights series ^1^. These articles are meant to disseminate recent scientific progress on Gilles de la Tourette Syndrome (TS). During each year, the article will be a work in progress, maintained as a web page on the Authorea online authoring platform (the working draft for 2016 appears here). After the calendar year ends, the article is finalized and submitted as the annual update for the Tics channel on F1000Research ^2^.

## Methods

We searched PubMed on 22 Jan 2016 using the search strategy: (“Tic Disorders”[MeSH] OR Tourette NOT Tourette[AU]) AND 2015[PDAT]. This search returned 202 citations and includes articles appearing online in 2015 but not officially published by year end (from journals that still focus on the paper user interface). We also reviewed F1000Prime recommendations and presentations of interest at selected medical conferences. Articles were chosen based on a purely subjective assessment of interest, guided by our judgment of possible future impact on the field.

## Results

### Phenomenology and natural history

***Tic suppression*.** Since tic suppression is part of the treatment protocol for the Comprehensive Behavioral Intervention for Tics (CBIT) and for Exposure and Response Prevention, there has been increased interest in investigating the characteristics of tic suppression and the factors that affect it. A study of 26 TS adolescents compared free ticcing with a tic suppression condition ^3^. During the free ticcing condition, tic distribution across body locations was consistent with the view that most tics occur at the level of the shoulders and above: eye tics were the most frequent, followed by facial/cervical tics, and those involving the arms and legs. Tics involving the trunk were the least common. During the tic suppression condition, eye tics increased in 10 subjects, as did hand tics in 3 subjects. Tic suppression was most successful for tics in body locations generally associated with fewer tics, such as the legs and trunk. The authors suggest that tic suppression involves specific, rather than global, inhibition since some types of tics were easier to suppress than others. Historically, other categories have been used to classify tics, such as simple vs. complex tics and motor vs. phonic tics. The results of this study suggest that future research may benefit from including body location in tic analyses.

By definition, children with Tourette syndrome (TS) have had tics for over a year. They can often suppress their tics briefly and they do so more effectively when rewarded for successful suppression. It has not been known whether the ability to suppress tics develops only with practice over the years of having tics or whether the ability to suppress tics is present when tics initially occur. Greene and colleagues addressed this question in children whose tics had developed within the past few months ^4^. When children received tokens with monetary value for tic-free intervals, they had significantly more of these intervals compared to a baseline, unrewarded condition. This result suggests the possibility that behavior therapy for tics may work, at least for some children, even before TS can be diagnosed.

Another study examined the association between tic suppression and quality of life. Although most tic patients frequently try to suppress tics, they find suppressing them uncomfortable and distracting. However, those patients who are more satisfied with their ability to suppress their tics also report a higher quality of life ^5^.

***Sensory phenomena*.** Premonitory urges are common in TS, are perceived as triggers for tics, and are as bothersome as the tics themselves to many people with TS. Premonitory urges have a sensory component and many TS patients also report sensory sensitivities. Researchers have been clarifying the nature of premonitory urges and attempting to determine the underlying causes of sensory sensitivities.

Scores on the Premonitory Urge for Tics Scale (PUTS) and the University of São Paulo Sensory Phenomena Scale (USP-SPS) were significantly correlated with total tic severity, tic complexity and vocal tic scores in youth and adults with TS ^6^. The PUTS and USP-SPS scores were also correlated with scores on the Dimensional Yale-Brown Obsessive-Compulsive Scale. This study provides additional evidence that the association between premonitory urges and tics is complex and may be influenced by obsessive-compulsive tendencies.

Another study examined the association between premonitory urges and interoceptive awareness ^7^. Interoceptive awareness was measured by how well subjects were able to mentally track their heartbeats, without being able to take their own pulse, during a specific period of time. Interoceptive awareness, tic severity, and obsessive-compulsive symptom severity were used in a multiple regression to predict PUTS scores. Greater interoceptive awareness and tic severity were significantly associated with higher PUTS scores while OCD symptom severity was not. The authors suggest that high interoceptive awareness may lead people to set a low threshold for perception of their own internal physiological sensations and therefore cause them to interpret these sensations as an urge to tic. Interestingly, TS subjects had lower interoceptive awareness than controls overall, suggesting that this downregulation of interoception might reflect a compensatory process.

***Symptoms and comorbidity*.** Recent research has again demonstrated the wide prevalence of TS-associated comorbidities and is a reminder of the need to perform studies with large enough sample sizes to examine the effects of comorbidities on the dependent variables of interest.

A retrospective review of 1,000,000 people in the Taiwan National Health Insurance Research Database examined the association between epilepsy and TS ^8^. 1,062 children and adolescents with TS were matched on age and sex with a control group of 3,186 subjects. The TS group had an 18-fold increased risk of epilepsy compared to the control group; the risk was still elevated 16-fold after adjusting for comorbidities ( *i.e.*, bipolar disorder, depression, learning difficulties, autism, anxiety disorders, sleep disorders). The authors acknowledge the possibility that some tics may have been mistaken for seizures, given the nature of the data set, but these data do suggest an important hypothesis for future confirmation.

In a large study of psychiatric comorbidity in TS, approximately 800 families were recruited primarily from TS specialty clinics in four different countries over a 16-year period ^9^. A total of 1374 participants with TS and 1142 family members unaffected by TS were included. 86% of the TS participants had at least one psychiatric comorbidity and 72% had either OCD or ADHD. Mood, anxiety and disruptive behavior disorders each occurred in approximately 30% of the TS participants. The genetic correlations between TS and mood were accounted for by ADHD and OCD, while ADHD alone accounted for the genetic correlations of TS with anxiety and disruptive behavior disorders.

A study of 400 patients seen at a TS specialty clinic found that 39% had coprolalia and 20% had copropraxia ^10^. When the 222 patients with full comorbidity data were examined, only 13.5% had “pure” TS ( *i.e.*, without comorbidities). None of the “pure” TS group had coprolalia and none had a family history of obsessive-compulsive disorder.

Emotional regulation difficulties were described in three studies, reminding us that for many TS patients, tics are not their most problematic symptoms. Greater irritability was seen in TS adults with more severe tics and those with comorbid ADHD ^11^. Eddy *et al.* found that both male and female TS subjects, compared to controls, reported more distress during emotionally intense situations and rated their abilities to take other people’s perspectives lower ^12^. An experienced clinician who has done research on “rage attacks” in TS has provided a clinically useful summary of current knowledge regarding aggressive symptoms in TS, OCD, ADHD and mood disorders, and described treatment options ^13^. Given that emotional regulation difficulties are frequently associated with greater tic severity, improving emotional modulation skills may be an appropriate target of psychological interventions.

More research is also being conducted on personality differences associated with TS. A small study of 17 male adolescents ^14^ found that the only significant difference between the TS subjects and 51 age- and gender-matched controls on the Minnesota Multiphasic Personality Inventory-Adolescent version was that the TS subjects scored higher on the Obsessiveness Content Scale. In contrast, a study of 50 TS adults in Germany used a variety of instruments to measure psychological symptoms and personality traits ^15^. Comorbidities were common (41% OCD, 28% depression, 26% ADHD). Patients with OCD had more severe tics and there was a trend for those with ADHD to have more severe tics. Only 29% of the patients had no pathological personality traits, as measured by the Inventory of Clinical Personality Accentuations. The demand-anxious trait was the most common personality trait seen in patients and was present in 39%, while histrionic personality traits were not found in any patients. Personality traits in patients with “pure” TS were comparable to those of the control group. Interestingly, ADHD did not contribute to increased probability of pathological personality traits. Although quality of life was affected by both personality traits and comorbidities, personality traits had a larger impact on quality of life.

***Course*.** More research is being conducted on the developmental progression of tics and other symptoms in TS. This work may provide clues that help clarify what factors contribute to the appearance and disappearance of transient tics and what factors explain the disappearance or amelioration of tics as children with TS enter adulthood.

One study examined home videos recorded in the first 6 months of life from 34 children who were exhibiting autistic behaviors in their second year of life ^16^. Families reported that development during the first year of life had been normal. Videos of 18 boys were examined in detail. The primary focus of the study was on autism, though 11 of the 18 subjects were later diagnosed with TS. The nearly ubiquitous availability of home baby videos in some cultures suggests that a similar pseudo-prospective study design could be used to identify behavioral features predicting later development of TS.

The clinical characteristics of children who developed TS before the age of 4 were compared with those whose tics developed at age 6 or older ^17^. The younger group had a higher rate of speech dysfluencies ( *e.g.*, stuttering) and oppositional defiant disorder. There was no difference between the two groups in prevalence of ADHD or obsessive-compulsive symptoms. Interestingly, the children in the early-onset group were more likely to have mothers with tics. The authors attributed this to mothers with tics being more likely to recognize tics in their children. The authors also suggest that prenatal or perinatal maternal environmental factors may contribute to the development of tics. An alternative explanation may relate to the fact that TS is much less common in girls than in boys. Consequently, tics in a woman may represent a higher genetic load, resulting in a more severe form of tics and an earlier age of onset in her children.

Researchers re-evaluated 75 patients previously seen at a university-based TS clinic after a mean follow-up of 9 years ^18^. Reported TS impairment was more likely to decrease over time in males and increase in females. In adulthood, women were more likely than men to have an expansion of the number of body regions exhibiting tics, primarily in the upper extremities. This result suggests that sex continues to influence TS symptoms beyond adolescence.

### Etiology

***Genetics*.** The most important genetics news of the year may have been the presentation by Huang and colleagues at the Tourette World Congress in London in June, 2015, reporting on a large collaborative study of copy number variants (CNVs) in approximately 2,500 TS cases and 3,500 controls ^19^. They first tested some CNVs previously reported in various neurological and psychiatric illnesses; the most significant confirmation in this sample was of exonic deletions in *NRXN1*, a gene previously implicated in autism and TS ^20^. They also searched for new, large CNVs and identified a novel TS locus, *CNTN6*, that was significant at a whole-genome level by permutation testing. This gene and 4 of the 32 next most likely candidates this analysis identified are neural adhesion molecules ^20^. *CNTN6* is a reasonable candidate for etiologically contributing to TS: its expression in the brain varies during development ^21^, knockout mice show motor deficits ^22^, and an independent study found a variety of neurological and psychiatric symptoms (including 2 with OCD) in 14 patients identified by *CNTN6* CNVs ^23^. On the other hand, fewer than 1% of TS cases in this sample had CNVs in *CNTN6*, so its overall importance in TS remains to be more fully characterized.

A large collaborative group ^24^ studied *AADAC*, the gene encoding arylacetamide deacetylase, in which microdeletions had been identified in a previous, smaller copy number variation study. The authors provide evidence from several sources supporting the connection of *AADAC* and TS, including a significant overall association, new patients with *AADAC* deletions, and evidence that *AADAC* is expressed in the brain, though most strongly in cerebellum, hippocampus and olfactory bulb, rather than the basal ganglia.

A collaborative genetic study ^25^ demonstrated an association of TS with 33 genes related to glycolysis or glutamine metabolism. None of the individual genes would have survived correction for multiple testing and the results were consistent with a combined effect of many genetic variants of small effect. These results suggest a new direction for future genetic, electrophysiological, imaging and pharmacological studies.

Yu *et al.* reported a genome-wide association study (GWAS) from 1,310 people with OCD, 834 with Tourette syndrome, 579 with both OCD and a chronic tic disorder, and over 5,500 controls matched for ancestry ^26^. A significant polygenic component was identified for OCD without tics, but not for the combined patient group or other subgroups. Overall, this study is consistent with previous work but it provided disappointingly few novel results.

An international study examined tic symptoms in the United States and the Netherlands ^27^. Three factors (complex vocal tics and obscene behavior, body tics, and head/neck tics) accounted for 49% of the variance in tic-related symptoms. There was no evidence of heritability for the second factor, but the h2r was approximately 0.2 for the first and third factors when age and sex were included as covariates. Heritability for these narrower tic phenotypes is considerably lower than the heritability estimates (up to 0.65) when comorbid conditions such as OCD and ADHD are included. These authors conclude that broader tic phenotypes, rather than narrower pure tic phenotypes, may be more successful at identifying the genetic mechanisms underlying TS.

***Environmental risk factors*.** Researchers used data from the Avon Longitudinal Study of Parents and Children to identify maternal factors that increase the risk of tic disorders in offspring ^28^. The Avon Longitudinal Study is an ongoing, prospective, pre-birth cohort study of all children born in Avon, United Kingdom, between April 1, 1991, and December 31, 1992. Maternal questionnaires were administered throughout pregnancy and mothers also completed questionnaires about themselves and their children’s development every 6 months from the child’s birth to the age of 7 and then yearly thereafter. In the final multivariate model, chronic maternal anxiety, evident both before and after the child’s birth, was associated with TS or chronic tic disorder in children. This association may reflect shared genetic susceptibility or prenatal exposure.

### Pathophysiology

***Pathological studies*.** An important study follows up on the autopsy results from the Vaccarino lab by comparing RNA transcripts from the basal ganglia of 9 TS and 9 matched control subjects ^29^. The most strongly associated set of downregulated transcripts involved striatal interneurons, consistent with the autopsy studies. The leading set of upregulated transcripts involved immune-related genes even though none of the TS subjects met proposed diagnostic criteria for PANDAS or PANS. The results obtained in the present study using brain tissue did not overlap with those of previous studies using blood samples. The authors interpret their results as implicating disrupted basal ganglia interneuron signaling in the pathophysiology of severe TS.

***Animal models*.** Rodent and monkey tic models have been developed in order to study tic generation mechanisms more directly and a number of studies using rodent models were published in 2015. Removing about half of the cholinergic interneurons in the dorsolateral striatum produced increased fragmented grooming behavior in response to repeated unpredictable acoustic startle stimuli in mice and also increased repetitive sniffing in response to D-amphetamine challenge ^30^. Ablation in the dorsomedial striatum did not produce similar deficits. None of the experimental conditions produced a change in prepulse inhibition. These results provide partial support for the autopsy data linking some characteristic TS symptoms to cholinergic interneuron deficits in the dorsolateral striatum.

A rat model was used to determine to what extent cortical and striatal input affected the temporal and spatial properties of motor tics ^31^. Focal blockade of GABA-A receptors with bicuculline injections into the anterior striatal motor region produced focal tic-like movements of the forelimbs. Medium spiny neurons (MSNs) and fast spiking interneurons (FSIs) exhibited increased activity during these movements, and all of the MSNs were only active during them. All of the FSIs were active during tics, but a minority followed this increase in activity by a decrease. Four different patterns were seen in globus pallidus (GP) neurons. About half of the GP neurons demonstrated increased activity during the tic-like movements, while the rest showed only inhibition or a combination of inhibition and excitation. The effects of cortical input were studied using short bursts of high-frequency electrical pulses applied at random intervals to the region of primary motor cortex representing the forelimb. Stimulation was provided before and after the bicuculline injections. The results suggested that the precise timing of tic occurrence was related to both incoming excitatory cortical input and the delay since the previous tic. These results support the fundamental involvement of the corticostriatal network with tic occurrence.

The role of GABA in tic generation was also studied in adult mice by injecting the GABA-A antagonist picrotoxin into areas throughout the cortex and striatum ^32^. Infusions into the central and dorsolateral striatum produced tic-like movements of the front paw, hind paw or head. Infusions into the dorsomedial striatum did not have a significant behavioral effect. Infusion into the ventral striatum produced increased locomotor activation in addition to sterotypical sniffing and wall licking without other tic-like movements. Infusions into the sensorimotor cortex produced tic-like movements in addition to increased behavioral activation involving cage exploration, sniffing, and occasional licking. When an NMDA receptor antagonist was infused into the dorsolateral striatum prior to infusing picrotoxin into the same location, tic frequency decreased significantly, thus demonstrating a role of glutamatergic activity in tic generation. Infusion of a GABA-A agonist into the sensorimotor cortex prior to picrotoxin infusion in the dorsolateral striatum also resulted in significant tic suppression. EEG recordings ruled out seizure activity. The authors summarize these results as providing evidence that these tic-like movements require corticostriatal interactions, with a key role for glutatmateric afferents, rather than autonomous striatal activity.

In a genetically engineered mouse model of TS+OCD (“Ticcy” D1CT-7 transgenic mice) ^33^, a small population of dopamine D1 receptor-expressing (D1+) somatosensory cortical and limbic neurons is chronically potentiated, resulting in cortical and amygdalar glutamatergic excitation of striatothalamic, striatopallidal and nigrostriatal subcircuits. Tics were decreased by the use of drugs that acted at different points in this “hyperglutamergic cortico/amygdalo-striato-thalamo-cortical [CSTC] circuit”. Excitatory forebrain serotonin and norepinephrine activity was blocked by ritanserin (a serotonin 2a/2c antagonist) and prazosin (an *α*_1_ adrenergic antagonist) respectively. In contrast, downstream striatothalamic neurons’ glutamate-triggered GABA output and downstream nigrostriatal neurons’ glutamate-triggered co-modulatory dopamine output were blocked by moxonidine (an agmatine/imidazoline-1 agonist) and bromocriptine (a D2 dopamine agonist) respectively. All four of these drugs decreased tic frequency and were considered to be “circuit-breakers” for the hyperglutamatergic CSTC circuit, thus supporting an important role of glutamate in generating the abnormal tic-like movements seen in these mice.

***Neuroimaging studies*.** The challenges of using neuroimaging techniques to study pediatric and clinical subjects are described in detail along with suggestions concerning various strategies that can be used to collect higher quality data ^34^. The profound effects of even very small head movements on structural MRI analyses were identified in a well-designed study ^35^. T1-weighted MRI of brain was acquired in 12 healthy adults while they were still or engaged in specific types of movement including nodding, head shaking or a movement each subject invented and then repeated during the scan run. Even during scans when subjects attempted to remain still, there was an average of 3 mm/min of accumulated motion measured as root mean square displacement per minute. During the motion conditions, substantial impact was found on gray matter volume and thickness estimates. Apparent volume loss averaged approximately 0.7% mm/min of subject motion. The greatest reductions in gray matter occurred in pre- and post-central cortex and in the temporal lobes. Motion-associated increases in thickness were seen in some frontal regions and deep sulci such as the medial orbital frontal region. Significant effects due to motion were still present even after excluding scans that failed a rigorous quality control procedure. Recommendations included reducing head motion during scans as much as possible, controlling for motion in statistical analyses, and using correlational analyses to determine the associations between head motion and the predictors of interest. Tisdall and colleagues described a method to limit the effects of movement artifacts by using a motion tracking system to provide prospective motion correction during scanning ^36^.

A whole-brain analysis of cortical gray matter found reduced gray matter (GM) thickness in the insula and sensorimotor cortex for 29 TS children and young adults compared to a matched control group ^37^. GM thickness in these areas correlated negatively with Premonitory Urge for Tics Scale scores.

Resting-state functional magnetic resonance imaging identified greater functional connectivity between the right dorsal anterior insula (dAI) and the bilateral supplementary motor area (SMA) in TS adults compared to controls ^38^. Post-hoc analyses found significant correlations between PUTS scores and connectivity between right dAI and right SMA2 and between right dAI and left SMA1. These regions may be involved in the increased awareness of body sensations that tend to be associated with premonitory urges. The authors paid attention to head movement and removed high-movement frames. However, recent result suggest that the motion threshold of 0.4mm used in this analysis, and the choice not to regress global signal, may not adequately remove artifactual correlations between brain regions due to residual small head movements during the scan ^39^.

A review summarized TS task-based fMRI studies in TS including studies of tic suppression, voluntary motor execution, voluntary motor inhibition, and tic severity ^40^. Free ticcing conditions (four studies) most commonly activated the left cerebellum, right cingulum, left middle frontal gyrus, the Rolandic operculum, right pallidum, right SMA and thalamus. In motor response inhibition studies, on No-Go trials TS subjects exhibited greater activation in the bilateral prefrontal cortex, thalamus and caudate. In contrast, on voluntary motor execution tasks greater activation in TS subjects was seen in the left prefrontal cortex, right cingulum, and the anterior SMA. Tic severity ratings were correlated with greater activation of the right dorsal premotor cortex and the SMA. Anterior cingulate cortex and SMA were involved across task types. The thalamus was involved in all types of studies except for self-produced movements. The authors also briefly summarize the many issues related to neuroimaging tasks, such as associated comorbidities, medication effects, the need for longitudinal studies, and the confounding effect of tics during scanning. Additional neuroimaging studies of note are noted in Table 1.

**Table 1.** Additional neuroimaging and electrophysiology studies.

Reference	Comment
Motor execution and motor imagery ^41^	An exploratory study found increased cortical premotor and prefrontal neural activation for both imagined and performed movements in TS subjects compared to controls. Premotor activation during the motor imagery task was correlated with tic severity.
Structural MRI in pediatric TS ^42^	A preliminary report of a multi-site study with over 200 subjects found TS children had greater gray matter volume in the posterior thalamus, hypothalamus and midbrain in addition to decreased white matter volume in orbital prefrontal and anterior cingulate cortex.
DTI and the corpus callosum ^43^	Axial diffusivity (AD) was reduced in treatment-naive boys with “pure TS” compared to controls. AD was negatively correlated with tic severity, although this result was not significant after Bonferroni correction.
TS and Chronic Tic Disorder ^44^	Both TS and chronic tic disorder groups exhibited similar increases in parietal and central event- related potentials, adding to evidence that TS and CTD may be the same illness.
Event-related potentials ^45^	Reduced P300 amplitudes in frontal regions were related to TS-associated comorbidities rather than to TS itself.

***Electrophysiology*.** Local field potentials associated with spontaneous tics were studied in 3 patients during DBS surgery ^46^. In all 3 patients repetitive thalamo-cortical coherent activity was present from 800 to 1500 msec prior to tic-associated muscle contractions. The frequency range affected varied among the patients and there were also ongoing intermittent intra-thalamic coherences that were not synchronized to the tics. The authors speculated that specific DBS targets may not matter as much as whether the target is part of the striato-pallido-thalamo-cortical network. However, since these patients were older and had very severe and complex tics, the authors acknowledge that it is not yet clear to what extent these results generalize to the TS population as a whole. Additional studies are noted in Table 1.

***Pharmacological studies*.** GABA involvement was studied in 23 TS children, aged 8–12, and 67 controls using a battery of vibrotactile tasks with a subset of the children (19 with TS, 25 controls) also undergoing GABA-edited magnetic resonance spectroscopy (MRS) ^47^. Lower GABA concentration in the right sensorimotor cortex correlated with greater motor tic severity ( *r* = *–*0.55). There were no significant differences between groups on reaction time and baseline amplitude discrimination threshold. However, TS children showed impaired tactile adaptation. The authors suggest that MRS GABA and tactile measures might useful as biomarkers of treatment response.

Positron emission tomography (PET) was used to investigate striatal D2/D3 dopamine receptor availability in TS subjects and controls using a D2/D3 receptor antagonist ([ ^11^ *C*]raclopride) and an agonist with preferential binding to D3Rs ([ ^11^ *C*](+)PHNO) ^48^. No differences were found in striatal regions when the TS subjects were compared with the controls, and there were no significant correlations between receptor availability and tic severity. The authors concluded that their results challenge the widely held view that striatal dopamine receptors have a fundamental role in TS pathophysiology, though they acknowledged that endogenous dopamine levels may have influenced the results since these radiotracers are displaceable by physiological synaptic concentrations of dopamine.

Two studies examined dopamine D1 and D2-like receptors in healthy adults, measuring D1R and D2R availability during stop-signal and continuous performance tasks ^49^. Stop-signal reaction time was negatively correlated with both D1R and D2R receptor availability in the the associative and sensory motor regions of the striatum. In contrast, neither D1R nor D2R receptor availability was associated with performance on the continuous performance task, suggesting that stop-signal and continuous performance tasks are associated with different neurochemical mechanisms related to motor response inhibition. In a study of healthy adults, learning from positive outcomes was positively correlated with D1R binding in the putamen and caudate while there was an inverted U-shaped relationship ( *r* ^2^ = 0.19) between learning from negative outcomes and D2R binding in the putamen ^50^. A dietary manipulation that reduced dopamine precursor levels significantly improved learning from negative outcomes. These results were interpreted as providing evidence that dopamine acts as a reward prediction error signal rather than as a saliency signal.

A detailed review focuses on histaminergic modulation of striatal function and its possible role in TS ^51^. The authors suggest that during wakefulness and increased attention, histaminergic neurons will be more active with the result that the striatum will be more responsive to thalamostriatal input and feed-forward inhibition will dominate. Several lines of evidence related to the role of histamine in TS were discussed. A family linkage study identified a rare mutation in the gene encoding histidine decarboxylase. HDC transgenic mice exhibit decreased pre-pulse inhibition of startle responses and an increase in a variety of amphetamine-induced stereotypies that were prevented by pretreatment with histamine infusion or use of haloperidol. Reduced histamine production was suggested to produce dopaminergic disregulation of the basal ganglia and symptoms similar to those seen in TS.

***Clinical and neuropsychological studies*.** In an intriguing report from a group studying social cognition, TS subjects exhibited intact mentalizing when observing animated triangles demonstrating simple and complex interactions ^52^. However, unlike controls, TS subjects also tended to attribute human-like intentions when two triangles were moving randomly. This tendency was not explained by clinical symptoms or by other constructs such as executive function or alexithymia.

Two studies examined the effects of comorbidities on social and cognitive skills. Social responsiveness and cognitive flexibility were examined in TS children and adolescents ^53^. TS subjects were rated as having poorer social motivation and skills, using the Social Responsiveness Scale, compared to age-matched controls. TS subjects also took significantly longer to complete the Trail Making Tests which measure cognitive flexibility and visual motor integration. However, of the 31 TS subjects, 11 had OCD, 18 had ADHD and 8 had an anxiety disorder. Once these comorbidities were taken into account, group differences on the Trail Making Tests and the Social Responsiveness Scale were no longer significant. These findings demonstrate the need for studies to have adequate sample sizes to provide sufficient power to disentangle effects related specifically to tics rather than to other symptoms. Another study was designed to separate effects attributable to OCD ^54^. Sustained attention, using a continuous performance test, was examined in 48 children and adolescents who had OCD alone, tic disorders (TD) alone or both OCD and TD. A high rate of ADHD was seen in all groups (62% of the OCD+TD group, 27% in the TD alone group, and 20% in the OCD alone group). Anxiety was also frequent (77% in the OCD+TD group compared to 49% for the other two groups combined). The OCD+ TD group had more errors of omission and higher reaction time variability. These results of this study provide additional evidence that the OCD+TD phenotype is associated with more severe symptoms including attentional difficulties and symptoms of anxiety.

Two studies examined motor control. In a clever analysis of video recordings of the eyes ^55^, spontaneous blink rate, which is related to dopamine levels, was higher in children with TS than in controls both during task performance and during a rest period. In contrast, pupil diameter, which is related to norepinephrine levels, was correlated with anxiety in TS subjects although not in controls. Researchers also used a cognitive control task to measure ability to properly update current task information, ignore competing information when selecting between response options, and retrieve and use relevant response contingencies. Accuracy on this cognitive control task accounted for half of tic severity variance. In an unrelated study, TS children without ADHD or OCD had significantly greater difficulty maintaining postural stability than did age- and gender-matched controls, especially when subjects had access only to accurate vestibular, rather than visual or somatosensory, cues ^56^.

Somatosensory sensitivity was compared in adults with “pure” TS and controls by establishing thresholds for externally applied stimuli ^57^. No differences were found between the two groups, supporting the view that the sensory abnormalities seen in TS may be related to abnormal interoceptive awareness or abnormal central sensorimotor processing.

Three recent studies examined the effects of attention on tic frequency and the results have implications for how treatment protocols could be modified to increase effectiveness. In one study, the role of attention on tic frequency was examined under several conditions ^58^. Tic frequencies were lower for 12 TS subjects during a baseline condition when they were alone in a room compared to when they were alone in a room looking at themselves in a mirror. Researchers then determined whether the increase in frequency was due to increased attention to the tics themselves or due to increased self-awareness in general. In addition to the conditions previously described, 16 subjects were also shown videos of themselves while they were not ticcing. Tic frequency was again lower during the baseline compared to the mirror condition. Tic frequency was even lower when subjects were watching the video of themselves while not ticcing. The authors suggest that future treatments teach patients to attend to states when they experience fewer tics. Another study of TS adults ^59^ compared tic frequency while subjects were engaged in tasks that involved attending to particular fingers, colored circles, or whether a tic had occurred during a specific 2-second interval. Observations for these tasks were made both during free ticcing and tic suppression. Not surprisingly, more tics were seen during a baseline free ticcing condition. During the attention tasks, tic frequency was greatest while subjects focused on their tics. In contrast, tic frequency decreased during the color attention condition and decreased further during the finger attention condition. When subjects suppressed their tics, they reduced their baseline tic frequency similarly across all attention conditions. These results are consistent with the idea that internally-directed attention, especially with a focus on tics, may contribute to momentary increases in tic severity. The authors suggested that behavioral treatment might be more effective if it focused on teaching patients to focus on external events and voluntary actions when they are in situations that are most likely to result in ticcing. Anecdotal evidence has suggested that tics decrease when people are involved in musical activity, so Bodeck *et al.* systematically studied the effects of music. ^60^ Questionnaires completed by 29 patients supported the idea that listening to music and performing music decrease tic frequency. Eight TS subjects were then observed in a variety of conditions. Tics were almost completely eliminated when subjects were performing music. Listening to music and using mental imagery of musical performance also resulted in decreases in tic frequency. The authors suggested that focused attention, along with fine motor control and goal-directed behavior, produced the decrease in tics.

The stereotyped nature of tics has led some to suggest that the neural systems involved in habitual behavior may also be associated with tic generation. A complex, three-stage instrumental learning paradigm was used to compare medicated and unmedicated TS adults with a control group to determine whether they differed in goal-directed *vs.* habitual behavior ^61^. During the first stage, subjects learned to associate six different stimuli with six specific outcome pictures and a specific response (i.e., left or right key press). During the second stage, subjects were presented with two outcomes with an indication that one outcome was devalued (i.e., no longer associated with point rewards) and subjects had to press the key associated with the outcome that would still generate points. During the third stage ( *i.e.*, “slip-of-action” stage), the six outcomes were presented simultaneously with indications that two outcomes were devalued so that responding to the associated stimuli would no longer generate points. Subjects were instructed to press the key associated with stimuli associated with the still valued outcomes (i.e., “Go”) and withhold the response (i.e., “No-Go”) for stimuli associated with devalued outcomes. This task determined whether excessive “slips of action” were related to outcome devaluation insensitivity. A control Go/No-Go task, which involved devaluation of cueing stimuli, was used to measure to measure response rates where high rates on this task would indicate working memory deficits or deficient response inhibition. There were no group performance differences for the first two stages of the instrumental learning task or on the baseline Go/No-Go task. However, unmedicated patients showed a significantly higher response rate to devalued outcomes compared to controls (in Bonferroni-corrected *post hoc* analyses), while there was no difference between medicated subjects and controls. In addition, tic severity in unmedicated subjects was correlated with response rates to devalued outcomes and with stronger structural connectivity between the right supplementary motor cortex and the posterior putamen. The results obtained in this study contrasted with results on similar tasks obtained with subjects with obsessive-compulsive disorder without tics. The authors argued that over-reliance on habits in OCD without tics is associated with impaired knowledge of response-outcome associations, while this type of learning was intact in both TS groups in this study. They concluded that habit formation is enhanced in unmedicated TS subjects but medication may normalize responses.

### Treatment

***Psychotherapy*.** 2015 saw several practical advances in the psychotherapeutic treatments available for tics. TicHelper.com is a commercial adaptation of Comprehensive Behavioral Intervention for Tics (CBIT) to the Internet, discussed at the London congress in 2015. ^62^ It is potentially an important treatment option, especially for the many TS patients who do not live near a behavior therapist. Efficacy testing is ongoing (see the trial summary at ClinicalTrials.gov).

McGuire *et al.* dug into the data from 2 previously reported, pivotal, randomized controlled CBIT studies that together enrolled over 200 children and adults ^63^. The superior treatment benefit from CBIT, compared to a control therapy, could be attributed to differential improvement in only a few types of tics, including throat clearing, sniffing, and complex tics. In general, vocal tics were more likely to improve following CBIT treatment. The controlled breathing used as a competing response for vocal tics may have allowed patients to direct attention away from the associated premonitory urges in a way that muscle-tensing competing responses for motor tics did not. This report also extends previous information about premonitory phenomena, including varying prevalence of premonitory urges across specific tic types.

“Living with Tics” is a modularized cognitive-behavioral treatment focused on decreasing tic-related impairment and improving quality of life. This treatment program was recently tested in a randomized, wait-list control study, with the active intervention including up to 10 weekly sessions for children and adolescents ^64^. Treatment modules focused on a variety of themes including self-esteem, emotion regulation, parent training, cognitive restructuring, coping at school, overcoming tic-related avoidance, and 1 or 2 sessions of habit-reversal training. Active treatment led to improved child-rated quality of life and reduced blinded clinician-rated tic impairment compared to the wait-list control group. An additional 7 wait-listed youth then participated in the treatment program resulting in data on 19 participants for open-trial analyses. With the larger sample size the reductions in tic severity (i.e., 30%), anxiety, obsessive-compulsive symptoms, and parent-rated impairment were significant. Both the youth and their parents reported satisfaction with the intervention, serving as a reminder that improving quality of life can be a desired treatment goal.

A small, uncontrolled open trial of mindfulness-based stress reduction treatment involved 18 individuals who were at least 16 years old. ^65^ Treatment consisted of eight weekly two-hour group classes and one four-hour retreat. Participants were taught a tic-specific meditation exercise that involved noticing any urges to tic while maintaining a focus on one’s breathing rather than trying to change or eliminate the urge to tic. Only one subject dropped out and overall tic severity was decreased by 20% for participants who completed the program. An independent evaluator rated ten subjects as "much improved" or “very much improved” and these subjects were considered treatment responders. The gains for the fifteen participants who had not had medication changes in the interim were maintained at a one month follow-up visit.

An unblinded trial of psychotherapy using a cognitive psychophysiological model of tic behavior was conducted with 102 adults who had TS or chronic tic disorder ^66^. Ten weeks of individual psychotherapy involved a number of components including increasing tic awareness; improving muscle control; preventing excessive muscle tension; decreasing an overactive action style; identifying low and high risk activities in terms of tic probability; highlighting differences in behaviors, thoughts and feelings related to differences in tic probability; decreasing perfectionistic beliefs linked to tension; generalization; and relapse prevention. These psychotherapy components were chosen because prior research suggested that some people with tics have perfectionist beliefs leading to an “impulsive overactive style” that produces frustration and tension in addition to tics. Large effect sizes were seen for both patient groups compared to the waiting list control group: 65% of the chronic tic disorder group and 74% of the TS group had reductions of more than 35% on the Tourette Syndrome Global Scale (TSGS). YGTSS total tic scores were also significantly decreased for both patient groups. At the end of treatment 78 of 85 completers were rated as having no more than mild symptoms, regardless of the starting severity, while the other 7 were considered to have moderate symptoms. Large effect sizes were seen for tic subtypes ( *i.e.*, simple, complex, motor, phonic) and similar results were seen across tic body locations. The TSGS improvements were maintained in the 52 subjects who completed a 6-month follow-up evaluation.

A study by the same group examined the effects of this psychotherapy approach on event-related potentials and lateralized readiness potentials (LRPs) ^67^. EEGs were recorded for 20 TS subjects and 20 control subjects matched for age, sex and intelligence while performing a stimulus-response compatibility inhibition task. During the No-Go condition, the TS group exhibited a delayed and overactivated frontal late positive component on the No-Go portion of the task. The authors considered this frontal activation as evidence of an adaptive mechanism that allowed patients to perform similarly to the control subjects on the task. This difference did not normalize with psychotherapy, consistent with this interpretation. TS subjects exhibited a larger incorrect activation of the stimulus-locked LRP (sLRP) in addition to larger correct activation of the sLRP and delayed correct activation sLRP onset. Although sLRP onset and response-locked LRP (rLRP) peak normalized after psychotherapy, the larger sLRP amplitude did not. The authors suggested that therapy produced some normalization of activation in the premotor and motor cortex.

***Medication*.** Efforts to maximize the value of pharmacological treatment continue. Lemmon *et al.* reported on a carefully designed, thoughtful pilot study of glutamatergic modulators as tic treatment ^68^. Twenty-three children with TS completed a double-blind, parallel group study involving 6 weeks of placebo, D-serine (up to 30 mg/kg/day) or riluzole (up to 200 mg/day). YGTSS total tic scores improved by 25–38% in each group, without significant group differences. Although power was limited by the small sample size, this null result argues against eager pursuit of glutamatergic medications for TS at this juncture.

A meta-analysis of 22 randomized, controlled trials (RCTs) involving 2,385 children with ADHD found no causal relationship between stimulants and onset of tics ^69^. Rather, tics were associated with ADHD itself (5.7% in the psychostimulant groups and 6.5% in the placebo groups). This summary of previous evidence hopefully can further reassure patients and prescribers that stimulants do not cause tics. The evidence for this conclusion is strongest for methylphenidate (19 of the 22 trials), and in a large RCT in children with TS and ADHD, tics *improved* significantly with methylphenidate ^70^. In the meta-analysis, results were similar for the 3 trials involving amphetamines, but tic severity did increase in 12 adults with TS after a single intravenous dose of 0.3mg/kg D-amphetamine ^71^.

Four patients with treatment-refractory TS were studied in an early report of results using vigabatrin, a medication from the GABA-aminotransferase inhibitor class ^72^. One patient had a clinically significant reduction in tics while two others had tic reduction of approximately 25% but did not report subjective clinical improvement. Further study will be needed.

***Neurosurgery*.** A revised consensus statement on the use of DBS in TS appeared recently ^73^. It provides an important update to the 2006 recommendations ^74^, guided by almost a decade of generally positive results from an increasingly varied set of patients, though with limited evidence from randomized allocation treatment studies. Several major changes were made in the recommendations. First, the recommendation that DBS patients be 25 years or older has been replaced by a focus on clinical symptoms and severity. However, at a minimum, local ethics committee involvement was recommended for patients under 18 years of age. The second major modification in the guidelines was that a patient have a caregiver who would be available to accompany the patient to frequent follow-up appointments. In addition, the group recommended absence of active suicidal or homicidal ideation for 6 months prior to surgery. The final change in the guidelines recommended identifying and addressing personality disorders, malingering, factitious symptoms, embellishment, and other factors that can substantially complicate assessment and treatment.

Treatment effectiveness was examined by randomly allocated to DBS on-stimulation or off-stimulation conditions for the first 3 months after DBS leads were placed in the GPi (globus pallidus, pars interna). Patients were then switched to the other condition for another 3 months ^75^. Ratings were collected blind to stimulation status, and 13 patients completed assessments during both conditions. Total YGTSS scores were 15% lower at the end of the on-stimulation period compared with the off-stimulation period (p=0.048). Three serious adverse events occurred: two infections in the DBS hardware and one episode of hypomania. Further improvements were seen during the long-term open label treatment period. This study is important as it provides evidence of efficacy of stimulation *per se* from a study with random assignment to initial condition.

A case study raised the issue of temporary DBS treatment in a patient with TS and ADHD treated with thalamic DBS from age of 17 to 23 ^76^. This case highlights the issue of DBS in minors discussed in the consensus statement ^73^, but without adequate controls it is impossible to know whether DBS was the cause of tic improvement.

***Other treatment*.** Lisanby and colleagues reported a careful, randomized controlled trial of repetitive transcranial magnetic stimulation (rTMS) aimed at the supplementary motor area (SMA) in 20 adults with TS ^77^. There were suggestions of improvement, but on average, patients in the sham treatment group improved almost as much as those in the active treatment group. However, rTMS most effectively stimulates superficial regions of cortex, and an Israeli group employed a new coil designed to provide deeper stimulation in an open-label study of 12 patients with treatment-refractory TS ^78^. On average, the 12 patients did not improve significantly, but the treatment was well tolerated, and a *post hoc* analysis showed benefit in the 6 patients who also had OCD. A double blind, sham-controlled study in TS patients with OCD will be needed to confirm efficacy.

Cranial electrical stimulation (CES) was used in an open label trial to treat 42 children with TS who were less than 12 years old ^79^. Patients applied electrodes to their earlobes when they went to bed so that they could receive the treatment on a daily basis for 24 weeks. Treatment sessions lasted 60 minutes and children were allowed to sleep during treatment. Only one child dropped out before completing the study. The mean YGTSS score significantly decreased from 26 when they were initially seen to 11 after treatment completion. These results must be viewed as preliminary, since an RCT is required to rule out spontaneous improvement.

Another treatment undergoing initial efficacy testing is an oral orthotic device with which some patients have reported success. The rationale and design of the study, and initial safety results, were reported at the London meeting ^80^, and efficacy testing continues (see the trial summary at ClinicalTrials.gov).

### Tics, family and society

Two studies examined the association between parenting a TS child and parental stress. Using self-report data, Stewart and colleagues revealed that stress in parents of 74 children with Tourette syndrome was highly correlated with current ADHD symptom burden in the child ( *r* = .57) and, less strongly, with the child’s OCD symptoms ( *r* = .40) ^81^. However, perhaps surprising some readers, parental stress was not significantly correlated with current tic severity ( *r* = .18, *p* = .13). The correlation of parental stress with ADHD and OCD symptom severity was present to a similar degree in 48 control children without tics. These observations reinforce the need for optimal clinical management of TS to include appropriate treatment of associated comorbidities. In another study 28 parents of 21 TS children completed questionnaires on their anxiety, depression, and perceived stress ^82^. Eight of the parents reported a moderate or severe level of anxiety. In general, parents varied in terms of their self-reports of their coping skills and social support availability. These results remind clinicians that it is important to evaluate the social support available to families of TS children, and suggest that increasing parental coping skills may improve the quality of life in TS families.

Attitudes toward treatment were investigated using a survey of 295 parents of youth with TS and interviews with 42 young people with TS ^83^. These subjects were identified through Tourettes Action, a non-profit organization in the U.K. Patients tended to view aripiprazole more positively than other treatments. Three quarters of parents wanted access to behavior therapy for their child, but reported substantial trouble finding it. On the other hand, many young people were skeptical that behavior therapy would help. Importantly, patients identified several treatment outcomes other than tics *per se* as important to them: reduction of premonitory urges, increasing control over tics, and reducing anxiety.

### Additional reference sources

David Shprecher and colleagues recently published a thoughtful, forward-looking, but concise review of Tourette research and treatment ^84^. Mary Robertson ^85– 88^ reviews work that she and colleagues have done during her 35-year career treating TS patients and her 30 years of publishing TS research. Jankovic provided an overview of tic treatments ^89^, and one of our colleagues highly recommended a review of current medication treatment practice in Germany ^90^.

Jackson *et al.* describe how increased motor control occurs in TS adolescents along with a variety of compensatory mechanisms ^91^. These researchers make the case that increased inhibitory capacity within higher-order motor regions, such as the SMA, may alter motor excitability. Hawksley *et al.* summarize evidence suggesting that autonomic dysfunction may have a role in both epilepsy and Tourette Syndrome ^92^. They also discuss how electrodermal activity biofeedback reduced seizure activity significantly in patients with epilepsy. In contrast, TS subjects had difficulty reducing sympathetic activity, suggesting that treatment protocol modifications are needed for TS.

In December, 2015, a special issue on Tourette Syndrome appeared in the new journal *Current Developmental Disorders Reports*. Four of the 5 articles are clinically focused ^13, 93– 95^. The other article summarizes some of the recent advances in TS neuroimaging ^96^.

Abstracts of presentations from the 1st World Congress on Tourette Syndrome & Tic Disorders, London, 24–26 June 2015, are available online. The Tourette Association at regular intervals posts links on its web page to selected scientific articles relevant to TS ^97^. Finally, the authors are building the 2016 version of this article here, where readers are invited to submit suggestions or comments.

## Conclusions

TS is a complex condition to study because of the high rate of comorbidities, the differences in tic severity between people identified through community studies and those who participate in research studies conducted at tertiary care centers, the differences in types of tics (e.g., vocal *vs.* motor tics, simple tics *vs.* complex tics), childhood onset, and prominent fluctuations in severity over time. The studies published in 2015 demonstrate continued progress in addressing some of these obstacles.

Although many early research studies focused on the roles of dopamine and the basal ganglia in tic generation, more recent studies are adding to our understanding of the complexity of tic generation and identifying possible roles for glutamate and histamine, along with a variety of subcortical and cortical regions. A number of carefully executed, large treatment studies have been reported, yet none is effective for more than about half of the patients enrolled, and side effects, cost or availability limit many existing treatments. Novel treatments are still needed.
