# Supplementary material for: Tourette syndrome research highlights 2015
Source: F1000Res. 2016 Jun 24;5:1493. [Version 1] doi: 10.12688/f1000research.8769.1 (PMC4943293; doi:10.12688/f1000research.8769.1)
Supplement: Peer review history

# Peer review: Referee response for version 1

**DOI:** 10.5256/f1000research.9438.r14669

**Published:** 2016-07-12

**Authors:** Douglas Woods

**Affiliations:** 1 Department of Psychology, Marquette University, Milwaukee, WI, USA

This was a well-written overview of research published in the TS field during 2015.  I have no major concerns with the paper, but a couple of issues should be addressed in the paper.

1. The authors should make clear that this is not a methodological critique of any particular study or set of studies. It is simply an overview.
2. The authors should take care not to insert their subjective opinions into the paper. For example, on page 2, the authors note that "The most important genetic news of the year may have been...." I would like the readers to make that conclusion rather than the authors.
3. The authors conclusions about the effects of why vocal tics responded so well to CBIT are speculative (p. 8), and the authors should note that such an explanation would require confirmation.
4. At the end of the paper, I think the authors should make calls for additional studies. The 2015 year had things to say about why CBIT may or may not work, and the authors should call for additional work in that area. The authors should also call for large-scale translational studies that bring together various areas of basic science in the context of a clinical trial. This arrangement will lead to exciting new discoveries about tics and how treatments work. Finally, the authors should consider calling for research on optimizing treatment sequencing.

Overall, this was a useful review.

I have read this submission. I believe that I have an appropriate level of expertise to confirm that it is of an acceptable scientific standard.

# Peer review: Referee response for version 1

**DOI:** 10.5256/f1000research.9438.r14670

**Published:** 2016-07-12

**Authors:** Keith A. Coffman

**Affiliations:** 1 Division of Pediatric Neurology, Children's Mercy Hospital, Kansas City, MO, USA

I read, with great interest, the manuscript by Richards and Black. Overall, it was outstanding and provides an excellent, comprehensive review of the latest literature on Tourette Syndrome.

 I recommend that it be indexed, without revisions.

I have read this submission. I believe that I have an appropriate level of expertise to confirm that it is of an acceptable scientific standard.

# Peer review: Referee response for version 1

**DOI:** 10.5256/f1000research.9438.r14667

**Published:** 2016-07-06

**Authors:** Andreas Hartmann

**Affiliations:** 1 Department of Neurology, Assistance Publique- Hopitaux de Paris, Paris, France

A very comprehensive review of the TS literature in 2015 for which the authors must be thanked. Generally speaking, they provide a brief overview of the main results / points of each paper but do not dwell into interpretations and discussions, which is fine with me; all readers interested can look up the primary article. Also, there are no relevant articles missing from what I can gather as I have a weekly Pubcrawler alert for TS. The choice of papers is subjective, of course, and I was surprised not to find a few of our own group’s contributions, listed below, but I leave it up to the authors to elaborate on them or not.

I have read this submission. I believe that I have an appropriate level of expertise to confirm that it is of an acceptable scientific standard.

## References

[1] WorbeYLehericySHartmannA: Neuroimaging of tic genesis: Present status and future perspectives. *Mov Disord*.2015;30(9) : 10.1002/mds.26333 1179-83 10.1002/mds.26333 26377151

[2] MuellnerJDelmaireCValabrégueRSchüpbachMManginJFVidailhetMLehéricySHartmannAWorbeY: Altered structure of cortical sulci in gilles de la Tourette syndrome: Further support for abnormal brain development. *Mov Disord*.2015;30(5) : 10.1002/mds.26207 655-61 10.1002/mds.26207 25820811

[3] WorbeYMarrakchi-KacemLLecomteSValabregueRPouponFGuevaraPTucholkaAManginJFVidailhetMLehericySHartmannAPouponC: Altered structural connectivity of cortico-striato-pallido-thalamic networks in Gilles de la Tourette syndrome. *Brain*.2015;138(Pt 2) : 10.1093/brain/awu311 472-82 10.1093/brain/awu311 25392196PMC4306818
